# Preliminary characterization of biomolecular processes related to plasticity in *Acyrthosiphonpisum*

**DOI:** 10.1016/j.heliyon.2023.e23650

**Published:** 2023-12-13

**Authors:** Vincenzo De Fabrizio, Vincenzo Trotta, Luigi Pariti, Rosa Paola Radice, Giuseppe Martelli

**Affiliations:** a Department of Science, University of Basilicata, Viale dell’Ateneo Lucano, 10, 85100, Potenza, Italy; b School of Agricultural Forestry, Food and Environmental Sciences (SAFE), University of Basilicata, Viale dell’Ateneo Lucano, 10, 85100, Potenza, Italy; c Bioinnova srls, Via ponte nove luci, 22, 85100, Potenza, Italy

**Keywords:** Thermal stress, Aphids, Plasticity, Methylation process, Transposition process, Genome modification, Adaptation

## Abstract

Global warming strongly impacts many organisms' development, distribution and population structure. This problem has attracted the attention of many scientists to understand and study its actual effects, especially on insects influenced by environmental temperatures. Aphids are a model for studies of the genetics and physiology of stress. Aphids are characterized by parthenogenetic reproduction, which limits the effects of recombination on evolutionary processes, and have shown resistance to various biotic and abiotic stresses. This study was based on the hypothesis that aphids have optimized, over time, genetic mechanisms capable to give them plasticity through genome modifications mediated by transposition. To understand and evaluate the effects of heat stress, the expression levels of transposases and methylases were analyzed in mothers and daughters. Our results show that after four days from the thermal shock, methylation decreases in both mothers and daughters, while transposition significantly increases in daughters, thus generating gene variability, essential for adaptation.

## 1 Introduction

Temperature is one of the most important environmental factors affecting the life history traits of insects [[1], [2], [3]]. Insects could experience exposure to high temperatures throughout their development, changing their population dynamics [4,5]. Short extreme heat events (SEHEs) could occur in temperate zones in spring and summer [6]. SEHEs could have a negative impact on the biological performance and local persistence of short-lived species, such as poikilotherms [[7], [8], [9], [10], [11], [12]]. Extreme temperatures cause cell injury, which has a deleterious eﬀects on the physiology of an organism, especially on the metabolism [[13], [14], [15]]. In small poikilotherms, such as insects, a single exposure to very high temperatures could elevate body temperature to lethal levels and cause mortality. For example, the pea aphid *Acyrthosiphon pisum* cannot survive brief exposure to temperatures above 42 °C [16].

In response to sublethal environmental stresses, insects can generate novel phenotypes via epigenetic processes that, in turn, promote differences in gene expression allowing local adaptation [17]. The conversion of environmental stress experienced by parthenogenetic aphids into epigenetic information transmitted to the offspring is common, but until now these mechanisms have been poorly investigated.

In insects, heat shock leads to transcriptional reprogramming, throughout substantial changes in the transcription of the DNA methylation system, which affects the expression of different genes [18,19]. In animals, DNA methylation occurs primarily by the covalent addition of a methyl group to the 5′ positions of cytosine in a 5′-cytosine-phosphate-guanine-3′ dinucleotide context [[20], [21], [22]]. Although the differential methylation of a cytosine of the coding region in insect genes is less than that observed in mammals, the methylation in insects is an epigenetic mechanism that can regulate gene expression without direct modification of the DNA sequence [23]. Several mechanisms of DNA methylation have been identified in insects, ranging from low levels in fruit flies to very high levels in some specimens of cockroaches [21,24,25], suggesting a major role in insect evolution [26]. The aphid *A. pisum* has a DNA methylation system that includes homologs of all vertebrate DNA methyltransferases, and approximately 0.69 % of all *A. pisum* cytosines are methylated [23]. The *A. pisum* genome contains a complete set of DNA methylation genes, with homologs of two maintenance DNA methyltransferases (Dnmt1a and Dnmt1b), two de novo DNA methyltransferases (Dnmt3a and Dnmt3x) and only one Dnmt2. These data were common to other aphid species, such as *Diuraphis noxia* and numerous holometabolous insects [27,28].

Another enzyme that has been hypothesized to be responsible for the phenotypic plasticity of aphidsis the transposase. A coherent hypothesis is that aphids are able to adaptat to a stress by regulating a large group of transposable elements (TEs) and related genes [29]. Eukaryotic genomes contain extensive repeat units of TEs, or transposons, that are randomly distributed. For many of these repeats, the widespread distribution is due to transposition, a specific form of genetic recombination. Some of these elements move through a process known as conservation, in which the sequence of interest is excised from the original position and then reintegrated in a new site. Other mechanisms involve an increase in the number of copies of TEs since during this process the original element remains in its position, but one copy is inserted into a new one. Some plants have a unique insect protection known as HPR (or host-plant resistance), which is useful in managing insect infestations, particularly aphids [[30], [31], [32], [33]]. For example, the intensive use of pesticides against the infestation of the soybean aphid (*Aphis glicines*) has led to the selection of resistant individuals [34]. Also, more than 8 soybean genes, including RAG (Resistant to *Aphis glicines*) and HPR genes [35,36], are implicated in resistance to aphids, although the latter have developed resistance over time that can overcome host plant defenses [37,38]. Although aphids possess effector proteins that can evade or suppress the host plant defenses [[39], [40], [41]], it has been hypothesized that some genetic elements, such as TEs, could facilitate the adaptation process [42,43]. The increase in the mobility of TEs is plausible with the creation of a new genetic variability that is useful under particularly stressful environmental conditions [44]. Transposable elements generate gene variation, while the transcript expression of transposable elements can influence the expression of neighboring genes, that are important for adaptation [45,46]. The aim of this preliminary study was to evaluate and understand the possible mechanisms of genetic variability induced by thermal stress, focusing especially on methyltransferases and transposases, and how these mechanisms could vary in the successive generations capable of overcoming the stress. The results of the present study are useful for understanding how global warming affects the resistance and transgenerational plasticity of aphids at the biomolecular level.

## 2 Materials and methods

### 2.1 Insect rearing and heat shock treatment

A green clone of the pea aphid *A. pisum* was bred in the laboratory on broad bean plants (*Vicia faba* L.). The aphids were kept in a Binder KBF climatic chamber at 22 ± 1 °C and 85 ± 5 % relative humidity (RH), under an 18:6-h light/dark photoperiod. Broad bean plants of the variety “Aguadulce” were grown in pots (10 cm diameter), filled with commercial soil (COMPO SANA® Universal Potting Soil) in a greenhouse. In order to standardize the possible effects of the plant on the dynamics of the growth of aphids [47], young vegetative plants, with two well-developed pairs of leaves (3 weeks after sowing seeds) were selected. To eliminate maternal effects from the initial experimental individuals, groups of 60 virginoparae adult females were isolated from the aphid culture, placed on a fresh host plant maintained in a plastic box (20 × 15 × 30 cm height) and allowed to reproduce for 24 h in a Binder KBF climatic chamber. The adult aphids were then removed and the new born nymphs were reared to adulthood, producing the parental generation. To ensure that the results were not affected by host plant deterioration, 5-day-old aphids were transferred to a fresh plant and allowed to grow for a further 4 days. Since thermal resistance in aphids varies with age [48,49], cohorts of individuals of the same age were used in this work. 100 adult and virginoparae females of the parental generation were placed on a fresh potted broad bean plant and allowed to reproduce for 8 h under the above-mentioned conditions. The adult females were then removed. The new born nymphs were transferred on a new plant after 5 days and kept as a synchronous colony for 9 days, corresponding, at the rearing temperature of 22 °C, to the adult stage. However, before their manipulation in the experimental trials, the morphological characteristics of the aphid [50] were checked under a stereo microscope and the aphids that were not adults were discarded. Three synchronous colonies were used in each replicate. The study was conducted in three independent replicates for a total of 800 aphids. Adult aphids from one synchronous colony were collected and divided into 7 experimental groups of 20 aphids each. Five of these experimental groups were heat shocked and the remaining two groups were used as controls. Heat shock treatment was performed by placing the 20 aphids of each group in a Falcon® tube in a water bath (Argolab WB 12). Heat resistance was assessed by measuring aphid survival after exposure to 39.0 ± 0.2 °C (mean values ± accuracy) for 30 min. The thermal shock treatment was applied for 30 min, reducing both any heat hardening response [51] and preventing the starvation and desiccation of the treated individuals [1,49,52]. To assess the survival of the shocked aphids, they were then transferred to the base of a new young broad bean plant in a controlled temperature environment (22 ± 1 °C); the dead individuals were counted out of the total number of aphids transferred (the living ones climbed the plant and moved away from the base). Considering that many insects are usually immobilized for a short time immediately after the thermal shock, survival was checked 24 h after the treatment [49,51,53]. Aphid survival was also checked four days after the heat shock and estimated as the number of live aphids on the aphid that survived after one day. The control groups consisted of aphids that were subjected to the same manipulation, except for the heat shock exposure.

### 2.2 RNA extraction

For each replicate, aphids that survived the shock after one day and after four days, as well as their respective controls, were divided into two groups. Five aphids of each control group were directly placed into 200 μl of TBE Buffer (Tris-Borate-EDTA) 0.5 % and frozen. The aphids of the second group were placed individually on a microscope slide in a drop of TBE 0.5 % and, under a microscope, the abdomen of each individual was longitudinally dissected to remove the four largest embryos, corresponding to the stages 19–20 [54]. The aphid embryos were immediately placed in iced TBE 0,5 %.

The RNA from each group was extracted using NucleoZOL reagent (Macherey-Naghel). The protocol used was adapted and optimized for this matrix. The RNA was quantified using a NanoDrop™ 1000 spectrophotometer (NanoDrop Technologies, Inc., Wilmington, DE, USA). RNA was retrotranscribed in cDNA using FIRE Script® RT cDNA synthesis Mix (Solis Biodyne). For the qPCR assay, Power SYBR Green PCR Master Mix (Applied Biosystems®, Waltham, MA, USA) was used; the protocol is described in Table 1 and Table 2. The 7500 Fast Real-Time PCR System (Applied Biosystems) and 7500 Software v2.3 (Applied Biosystems) were employed. The real-time PCR primers were the following: 5′- TTTTTGGCGACTTGATATTGAC-3′ and 5′-ATTGAAGCAATATTTGGTTTGC-3’ (methyltransferase); 5′-TCAAGAAACTCTGTCGCCCG-3′ and 5′- ACGAATAAGGCAGTTTTTGACCT-3’ (transposase).

**Table 1:** qPCR protocol.

Amount	Reagent
5 μL	Power SYBR-Green PCR Master Mix (10X)
1.5 μL	Primer Forward 10 mM
1.5 μL	Primer Reverse 10 mM
1 μL	cDNA (10 ng/μL)
11 μL	Sterilised water

**Table 2:** qPCR amplification protocol.

Step	Temperature	Time
Holding Stage	95 °C	10 min
Cycling Stage (40 cycles)	95 °C	15 s
	60 °C	1 min
	72 °C	20 s
Melt Curve Stage	95 °C	15 s
	60 °C	1 min
	95 °C	30 s
	60 °C	15 s

The relative expression level was normalized by the expression of the housekeeping 18S mRNA in each sample, using the protocol described by Livak and Schmittgen [55].

### 2.3 Statistical analysis

As a binomial distribution is appropriate for modelling binary data (dead/alive), data on aphid survival after one day and after 4 days from the heat treatment were analyzed with two Generalized Linear Mixed Models (GLMM) with binomial errors and a logit link function fitted with REML (restricted maximum likelihood). In these models, “Treatment” (two levels, heat shock and control) is the fixed factor and “replicate nested within treatment” is the random effect. P‐values for the differences between treatments were obtained through ANOVA (type II Wald chi‐square tests).

The 2^−ΔΔC^_t_ method was used for the analysis of real-time PCR data and the groups not stressed were used as controls [55].

All statistical analyses in this study were performed using R-3.6.2 software [56] and Graph-Pad Prism 8.0.2 (San Diego, USA) [56].

## 3 Results

### 3.1 Heat shock survival

**Fig. 1:**
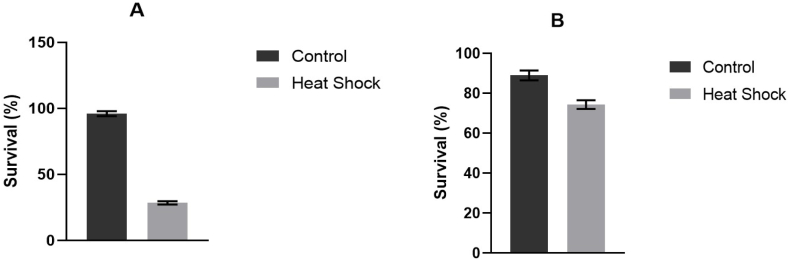
Mean values (± standard error) of aphid survival recorded one day (A) and between one and four days (B) after heat exposure at 39.0 ± 0.1 °C of the control and heat shocked aphids.

Fig. 1 shows (A) the mean values of aphid survival recorded one day and (B) between one and four days after the heat shock. Compared to control groups, heat shocked aphids had significantly lower survival after one day ($\chi_{\left(1\right)}^{2}$ = 75.9, *P* < 0.001). The reduced survival of aphids immediately after thermal stress reflects what has already been found in the literature [49] and indicates a reduced ability to trigger adaptation mechanisms in the short term. A significant reduction in survival was also observed in treated aphids between one and four days after the heat shock, although less pronounced ($\chi_{\left(1\right)}^{2}$ = 6.64, *P* < 0.01).

### 3.2 Gene expression

**Fig. 2:**
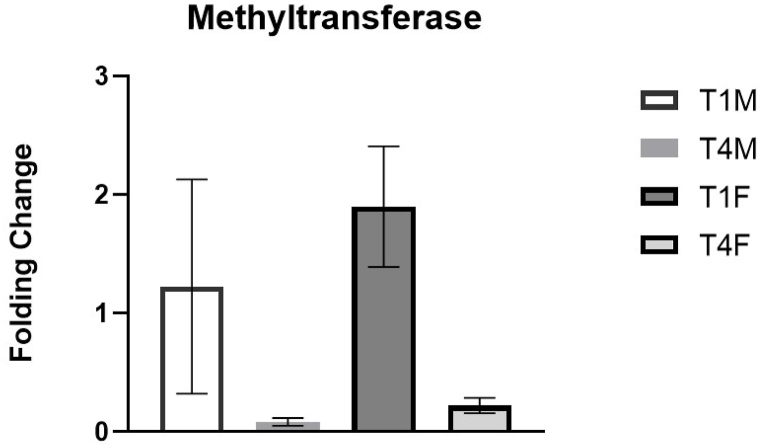
Methyltransferase expression level (2-^ΔΔCt^). T1M: Mothers treated day 1; T4M: Mothers treated day 4; T1F: Daughters treated day 1; T4F: Daughters treated day 4. (Data reported as Mean ± SD).

The expression levels of transposases and methyltransferases were analyzed. Fig. 2 shows the expression levels related to the methyltransferases in the heat-treated groups, normalized for the respective control groups. The heat stress determines an increase in the expression levels of this enzyme one day after the heat exposure. It is interesting to note that the expression level of methyltransferases in offspring is higher than in mothers, although not significant in relation to the range of variation highlighted. The expression levels of methyltransferases are close to zero (similar to the unstressed controls) in mothers and offspring 4 days after the treatment.

**Fig. 3:**
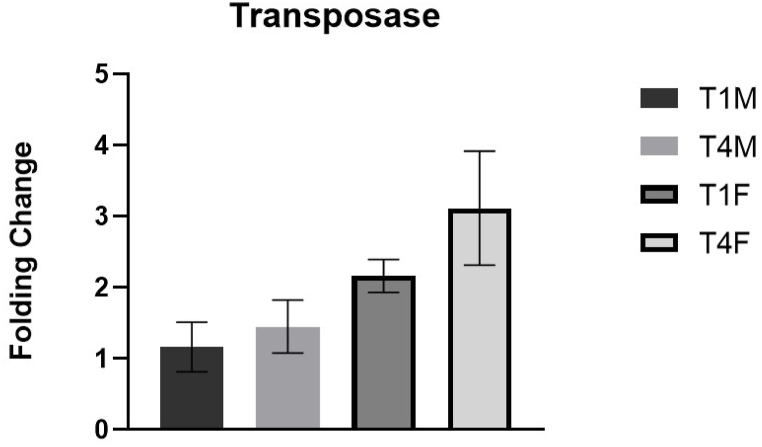
Transposase expression level (2-ΔΔCt). T1M: Mothers treated day 1; T4M: Mothers treated day 4; T1F: Daughters treated day 1; T4F: Daughters treated day 4. (Data reported as Mean ± SD).

It is evident that the treatment determines an extreme decrease in the expression levels of this enzyme after a few days. Comparing mother and offspring, the same trend is observed: after 4 days, methyltransferase is reduced in both mothers and offspring (p = 0.031). In particular, when referring to time 1, comparing mother and offspring, it is highlighted that the expression level of methyltransferases is higher in offspring than in mothers, although the data obtained do not appear to be significant (p = 0.155). In general, the expression levels of methyltransferases are close to zero (similar to the undressed controls) in mothers and offspring 4 days after treatment. A slight but not statistically significant increase in the expression level of transposase (Fig. 3) in the mothers is observed between T1M (1 day after the treatment) and T4M (4 days after the treatment). However, the comparative analysis of transposase expression levels in the offspring shows a consistent and significant increase when compared to the mothers, both at 1 day (p = 0.014) and 4 days (p = 0.031) after the treatment.

## 4 Discussion

Thermal shock is usually considered an abiotic stress that can significantly reduce the survival of insect populations [1,2,57]. The results of the present experiments showed that in the initial phase (after 1 day), the survival of heat shocked aphids was lower compared to the control group. This result indicates that the aphids are initially unable to activate those bio-molecular mechanisms that would allow them to fully overcome the stress [1]. After 4 days, the survival data are more encouraging, reaching a percentage value of approximatively 74 % in the treated group compared to the 89 % in the control group. These preliminary data have led us to hypothesize that the time required to induce variability within the offspring is longer than 24 h. To evaluate the gene expression of the methyltransferase and transposase and their impact on the survival of the mother and on the offspring adaptation [18,23,43,58], the RNA was extracted from both mothers and nymphs within the body of the mother, both in the heat-treated group and in the control group, that was used as a normalizer of the analysis, on day 1 and on day 4 after the stress. The results show how the level of methyltransferase expression is higher in the offspring than in the mothers 1 day after the heat shock. These data highlight how the treatment induces the implementation of a mechanism capable of generating genetic variability to realize different genomic structures in the offspring, able to respond to selective pressure and thus maintain a high level of adaptive fitness. This result leads us to hypothesize that the level of regulation of gene expression is initially higher in the offspring because subsequent generations have to implement mechanisms able to guarantee their better fitness. However, it should be noted that the levels of methyltransferases are reduced after four days in both mothers and offspring, because physiological modifications go beyond the adaptation phase to the selective pressure, allowing mechanisms capable of generating structural modifications. Indeed, four days after the heat treatment, the transposase level increased in both mothers and daughters. This result shows that the need to vary the parental genome structure by mobilizing TEs is essential to ensure survival and overcome the stress.

Overall, on a transcriptomic basis, it is possible to hypothesize the creation of a combined response to stress: close to the heat treatment, a quantitative/qualitative expansion of the gene products start, with a consequent increase in the level of bio-physiology activity. At the same time, when analyzing the level of expression of transposases, a higher level of expression is observed over time (1–4 days) and over generations (mothers and daughters).

The expression levels of methyltransferases lead us to hypothesize that an initial response on a physiological basis is followed by one that determines the creation of variability. In other words, it seems that fitness is first compensated by physiological adaptive mechanisms and then by the creation of new genomic structures (pro-evolutionary mechanism).

## 5 Conclusion

The physiology and the genetic mechanisms governing aphid response to stress and subsequent adaptation are highly complex. In particular, we have shown how the mechanisms underlying the transgenerational plasticity are articulated and involve bio-physiological mechanisms that at first sight, may appear non-specific and difficult to interpret. To the best of our knowledge, this is the first study demonstrating that aphids, and specifically *A. pisum*, initially respond to a selective pressure with an adaptive mechanism and, contextually, as the selective pressure persists, trigger a pro-evolutionary/pro-adaptive mechanism mediated by transposition.

This mechanism creates new variability that can confer a selective advantage (increase in fitness) to the offspring. This is very important considering that these insects mostly reproduce parthenogenetically and only during a short part of their annual cycle contemplates sexual reproduction as a mechanism useful for the creation of new genetic variability. Further studies should be conducted to better understand, and in more detail, the mechanisms underlying plasticity in aphids.

## CRediT authorship contribution statement

**Vincenzo De Fabrizio:** Formal analysis, Writing - original draft. **Vincenzo Trotta:** Methodology, Resources. **Luigi Pariti:** Resources, Writing - review & editing. **Rosa Paola Radice:** Formal analysis, Supervision, Writing - review & editing. **Giuseppe Martelli:** Conceptualization, Project administration.

## Declaration of competing interest

The authors declare that they have no known competing financial interests or personal relationships that could have appeared to influence the work reported in this paper.
